# Early Detection and Prevention of Age-Related Macular Degeneration

**DOI:** 10.7759/cureus.41169

**Published:** 2023-06-30

**Authors:** Gayathri Seluarize, Muhammad Najmi Khairudin, Fazliana Ismail

**Affiliations:** 1 Ophthalmology, Universiti Malaya, Kuala Lumpur, MYS; 2 Ophthalmology, Universiti Sains Islam Malaysia, Nilai, MYS

**Keywords:** wet age-related macular degeneration, blindness, elderly, vision loss, age-related macular degeneration

## Abstract

Age-related macular degeneration (ARMD) is a group of age-related changes in the macula that can be potentially vision-threatening. In the current era, there are options for treatment modalities that aim to preserve a patient’s vision. Poor vision not only serves as a significant factor in halting the elderly population from their daily activities, but it may also result in frequent falls, depression, and impairment of the ability to carry out activities of daily living. We would like to highlight in this clinical presentation simple tools for assessing the severity of disease and the importance of early detection of these patients.

## Introduction

Age-related macular degeneration (ARMD) is a leading cause of central visual loss among the elderly. The macula is an area in the retina that is responsible to maintain good central visual acuity. Central vision is essential as it determines the patient’s ability to perform near and distant activities comprising of basic daily living. ARMD causes severe visual loss and blindness ranging from 9% to 25% amongst patients aged 65-70 years worldwide [1].

ARMD can be classified broadly into dry and wet (exudative) forms. Dry ARMD, which accounts for 80%, is characterized by the presence of drusen at the macula and may progress to form geographical atrophy, which deems the patient with markedly reduced central vision.

Whereas neo-vascular ARMD accounts for only 20% of the total number of cases in which there is the presence of leakage from the choroidal neovascularization at the area of retinal pigment epithelium atrophy. This can present clinically as a submacular hemorrhage, edema, scarring, and fibrosis [2].

Treatment with intravitreal anti-vascular endothelial growth factor (anti-VEGF) has gained popularity in the management of ARMD cases. There are many large randomized control studies on the usage of intravitreal anti-VEGF injections. Concurrent treatment with photodynamic therapy has also been widely used as an adjunct in treating wet ARMD.

However, the power of identifying these patients who may benefit from all these available treatment options is in the hands of primary care practitioners.

In most instances, a complaint of blurring of vision in the elderly is attributed to cataracts, which are chronic and require a non-urgent referral or even treatment. On the other hand, ARMD cases need proper detection as timing and type of treatment determine visual outcomes. Detailed history taking on the presenting complaint may render an opportunity in identifying other potentially blinding conditions such as ARMD [3].

## Case presentation

Case 1: pigment epithelium detachment (PED)

A 76-year-old lady with underlying hypertension complained of painless central blurring of vision in her left eye. She had cataract surgery done in 2015 and maintained excellent postoperative vision.

**Figure 1:**
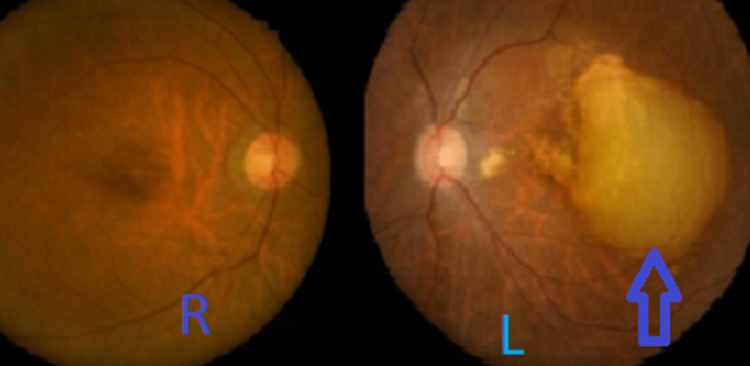
In this fundus photography, there is a large pigment epithelial detachment in the left eye.

On examination, the visual acuity of her right eye was 6/9 and her left eye was 6/12 (pinhole 6/9) with no relative afferent pupillary defect. The anterior segment of both eyes was unremarkable. The right eye fundus showed a pink disc, a cup disc ratio of 0.3, a normal macula, and a flat retina. The left eye showed a large pigment epithelial detachment with sub-retinal fluid at the macula as shown in Figure 1.

Ocular coherence tomography (OCT) macula showed sub-retinal fluid. Fundus fluorescein angiography (FFA) was performed and showed a pooling of fluid from the pigment epithelial detachment. An indocyanine green angiography (ICGA) showed a suspicious polyp.

She was subsequently given intravitreal anti-VEGF injections to halt the progression of the disease. Her vision remained at 6/10 for the left eye after a year of follow-up.

This patient had initially presented to the primary care clinic and was referred to an ophthalmologist promptly in view that she already had cataract surgery done. She received her first intravitreal anti-VEGF injection within a month.

Case 2: polypoidal choroidovasculopathy (PCV)

A 59-year-old gentleman with underlying diabetes and hypertension presented with left eye central scotoma for a few months.

On examination, his visual acuity of the right eye was 6/9 and his left eye was 6/30. There was no relative afferent pupillary defect. The anterior segment of both eyes was unremarkable. The right eye fundus showed a pink disc, a cup disc ratio of 0.3, the macula was normal, and a flat retina. The left eye had a large area of orange nodule at the macula and subretinal fluid.

OCT macula showed sub-retinal fluid on the left eye and FFA was performed, which showed an area of leakage at the superonasal to the fovea. An ICGA showed an area of leakage superonasal and temporal to the fovea.

Photodynamic therapy, which is verteporfin-guided laser photocoagulation, of the leakage area was performed and the patient was also given an intravitreal aflibercept injection.

His vision improved to 6/20 with a resolution of symptoms after a series of intravitreal anti-VEGF injections.

This patient was already under general ophthalmologist follow-up for annual screening for diabetic retinopathy. He was referred to the medical retina team and received his intravitreal injection after a month.

Case 3: macula scar

An 88-year-old gentleman with underlying hypertension and hyperlipidemia presented with complaints of left eye blurring of vision over the past three years. It was painless and gradually worsened over the years. He had already done cataract surgery 10 years ago and had good postoperative vision.

**Figure 2:**
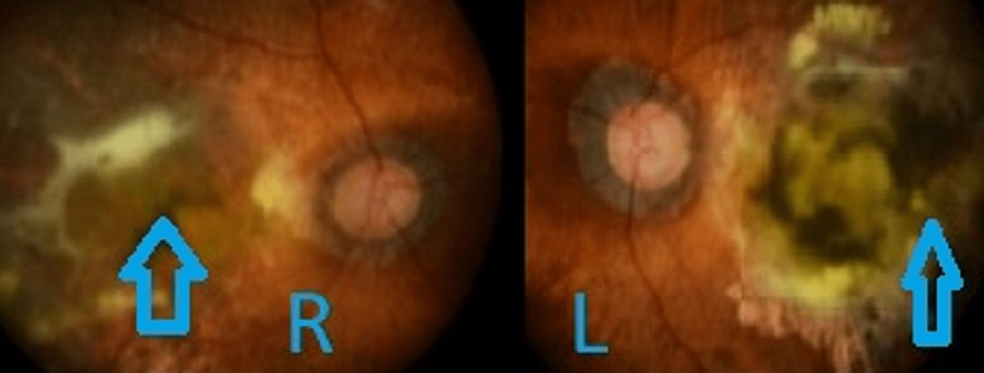
In this fundus photograph, a bilateral macula scar is present.

On examination, his vision for his right eye was counting fingers and his left eye was 1/60. Both eye fundus examinations showed extensive sub-retinal fibrosis at the macula, as shown in Figure 2.

OCT macula showed sub-retinal fibrosis and minimal intra-retinal cystic fluid. This patient was given trials of intravitreal anti-VEGF; however, there was no improvement in vision. His vision remained poor despite treatment in view of dense macula scarring.

This patient was also under general ophthalmologist follow-up and only received a referral to the medical retina team during his routine eye appointment. He received his first intravitreal injection six months after the onset of symptoms. He had earlier presented to primary care with similar complaints; however, there was no fundus photography done for him. Earlier detection of the disease by screening the fundus photography could have hastened the time to receive treatment.

Case 4: geographical atrophy

**Figure 3:**
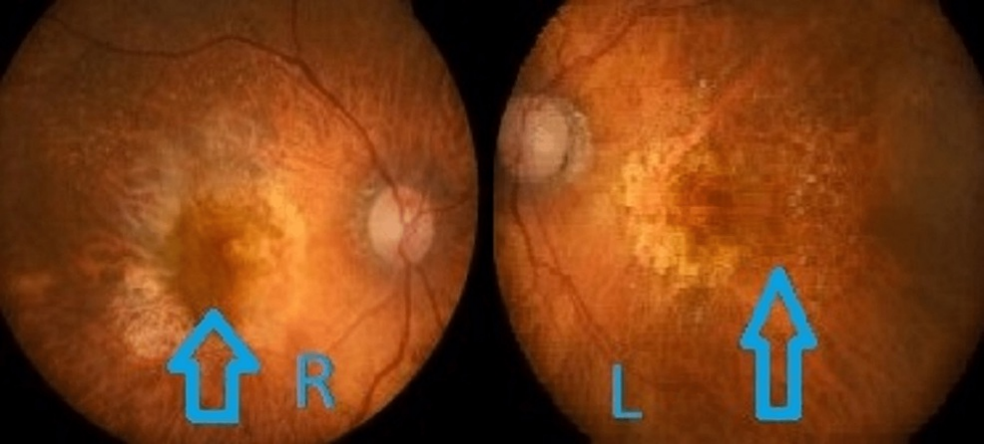
In this fundus photograph, geographical atrophy is seen in both macula

A 75-year-old lady with underlying dyslipidemia presented with bilateral eye blurring of vision for a year. Her vision for her right eye was 6/20 and her left eye vision was 6/30. Her fundus examination revealed the presence of bilateral eye geographical atrophy at the macula with multiple drusen at the parafoveal area, as shown in Figure 3.

OCT macula showed bilateral eye sub-retinal fibrosis and intra-retinal fluid. She was given multiple intravitreal injections in the past four years.

Currently, her vision has been maintained at baseline. She was referred promptly by primary care providers after reviewing the presence of macular fibrosis on the color fundus photography.

## Discussion

ARMD is a complex multi-factorial disease that ranks as one of the leading causes of blindness worldwide. As the world population ages, the prevalence is expected to rise in the near future with an estimation of cases predicted to reach 288 million by 2040 [4]. A population-based study conducted in Malaysia on data on prevalence and causative factors for blindness shows that 86.3% were avoidable blindness [5].

There are multiple risk factors that predispose to ARMD such as age, genetic predisposition, cigarette smoking, nutrition, dietary intake, exposure to sunlight, cardiovascular disease, and body mass index [6]. Identifying all the risk factors in an elderly patient with complaints of blurring vision will be crucial to increase the index of suspicion toward ARMD.

Patients with cataracts usually complain of gradual, generalized blurring of vision. They would describe their vision as cloudy and hazy. However, in ARMD, patients would have metamorphopsia and central scotoma. Therefore, primary care doctors are recommended to include these symptoms during history taking. It is recommended to identify patients with these complaints for referrals to ophthalmology. The Royal College of Ophthalmologists recommends commencing treatment within two weeks from the first presentation [7].

Fundus photography can be utilized to identify changes such as serous pigment epithelial detachments and sub-macular hemorrhages that may alert the receiving ophthalmologist for an urgent review. Screening for early changes in asymptomatic patients with high possible risk factors may help in preserving a better visual outcome. Visual acuity and the Amsler grid chart can also aid in this [8].

The mainstay treatment of ARMD is focused on managing wet ARMD, which is intravitreal anti-VEGF and photodynamic laser therapy. There are emerging studies that are being conducted in terms of cell-based and gene therapies for the treatment of dry ARMD and scarring. For advanced ARMD, patients can be offered low visual aid clinics, the use of magnifying glasses, or eccentric viewing techniques.

In Malaysia, general ophthalmology services are available nationwide at secondary and tertiary government hospitals. To treat wet ARMD effectively, patients need to be referred to the medical retina sub-specialty team for intravitreal anti-VEGF injection. Unfortunately, medical retina sub-specialty services are only available at a few centers and are running at very high loads. Accelerating the timing of referrals from primary care to ophthalmology for a complete assessment is paramount to shorten the interval from the onset of the complaint to the initiation of treatment by the medical retina team.

## Conclusions

Early detection and commencement of appropriate treatment by an ophthalmologist depending on the stage of the disease gives a better chance of vision improvement, preservation, and preventing or delaying total blindness. Primary care physician plays an important role to speed up the journey of the patient from first presentation to commencing treatment.
